# Invasive RNA Programs Optical Switching in DNA Nanoclusters for CRISPR Sensing

**DOI:** 10.1021/acs.nanolett.6c01946

**Published:** 2026-08-31

**Authors:** Reggie Gold, Yusha Imtiaz, Vinod Morya, Ken Halvorsen, Emmett Hanson, Mehmet V. Yigit

**Affiliations:** † Department of Chemistry, University at Albany, State University of New York, 1400 Washington Avenue, Albany, New York 12222, United States; ‡ The RNA Institute, University at Albany, State University of New York, 1400 Washington Avenue, Albany, New York 12222, United States

**Keywords:** fluorescence, CRISPR Cas12a, DNA nanotechnology, biosensing, nanoparticle

## Abstract

DNA-templated silver nanoclusters have regained attention due to recent advances in stability and tunable optical properties. Here, we report a highly regulated DNA-templated silver nanocluster (DFN2) that exhibits strong fluorescence at 561 nm and enables reversible, label-free fluorescence switching governed by nucleic acid hybridization. The fluorescence of DFN2 is efficiently quenched with complementary DNA or RNA and fully restored through invading RNA strand mediated displacement, enabling robust and programmable ON-OFF-ON optical control. We integrated this hybridization-responsive platform with CRISPR-Cas12a to construct a target-activated biosensing system. Upon recognition of a conserved genomic fragment from a foodborne pathogen *Listeria monocytogenes*, the activated Cas12a cleaves a regulator DNA, initiating a cascade of hybridization and strand-displacement reactions that restore nanocluster fluorescence through an invasive RNA. This strategy exhibits high specificity against conserved genomic regions from other foodborne pathogens. These findings establish DFN2 as programmable, enzyme-responsive optical reporters for rapid, sensitive, and modular point-of-care biosensing.

## Graphical abstract

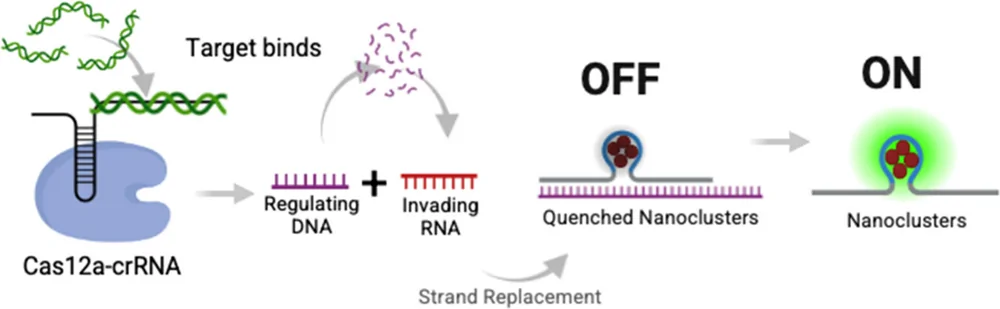

DNA-templated silver nanoclusters have recently attracted renewed attention, as emerging studies report improved stability and highly predictable responses to biological stimuli.^1,2^ These nanoclusters were long considered highly unpredictable, where even a single-nucleotide variation could significantly alter fluorescence properties such as excitation/emission wavelengths and long-term optical performance.^3–5^ Recent reports not only demonstrate more consistent formulations but also reveal increasingly predictable responses to designed biological interactions.^6,7^

Here, we report a functional DNA-templated silver nanocluster (DFN2) that exhibits strong fluorescence emission at 561 nm and undergoes fluorescence quenching upon hybridization with complementary RNA or DNA strands. Systematic evaluation of these nanoclusters demonstrates that their fluorescence is highly regulated and can be switched off via complementary “quencher” strands and subsequently restored using invading strands. This enables the rational design of switchable nanocluster-based systems for biosensing applications. Importantly, both the fluorescence switching and quenching mechanisms are entirely label-free, making these nanoclusters particularly attractive as RNA-regulated DNA nanomaterials with optical properties.

DNA and RNA nanotechnology are rapidly advancing, expanding the biological toolkit available for precision diagnostics and therapeutics at molecular level.^8–13^ Although RNA was historically considered an unstable molecule that limited its utility in *in vitro* systems, recent developments have demonstrated that RNA, alongside DNA, can serve as a robust building block for assembling functional biosystems^14–16^ and serve as a programmable regulators in various applications, including CRISPR-Cas12a-based detection platforms.

CRISPR-Cas12a has emerged as a powerful platform for nucleic acid detection due to its ability to precisely recognize conserved genetic sequences.^17,18^ Upon target binding, Cas12a undergoes activation and exhibits collateral cleavage activity toward surrounding single-stranded DNA (ssDNA), which can be harnessed for signal generation.^19–22^ By integrating this mechanism with secondary biological processes, detection sensitivity can be improved, and output signals can be more precisely controlled.^23–25^ In this work, we regulate the fluorescence properties of DNA-templated nanoclusters through an invading RNA molecule and integrate this response with CRISPR-Cas12a for biosensing applications. Specifically, we employ this cascade reaction to detect a conserved genomic region of *Listeria monocytogenes* and evaluate its specificity against Shiga toxin-producing *Escherichia coli* (STEC), *Campylobacter jejuni*, *Salmonella typhi*, and *Salmonella enterica*, all of which are common foodborne pathogens that pose major challenges to public health and the global economy.^26–30^

DNA-templated fluorescent nanoclusters were synthesized using a 27-nucleotide single-stranded DNA sequence (C6) containing a cytosine-rich region at its center, which is known to exhibit strong affinity for Ag^+^ ions, Figure 1a and Table S1.^31,32^ Incubation of this DNA template with silver ions in the presence of a reducing agent resulted in the formation of a strongly fluorescent DNA-silver nanocluster complex (DFN2), appearing as a characteristic light-brown colloidal suspension, Figure 1b. UV-vis absorbance measurements revealed a broad absorption band in the 300–400 nm range, Figure 1g. Fluorescence characterization showed an excitation at 471 nm, along with a strong emission peak centered at 561 nm, Figure 1h. Dynamic light scattering (DLS) measurements indicated a hydrodynamic diameter of approximately 210 nm, (Figure 1c), which is larger than expected for DNA-templated silver nanoclusters. To verify the size of the DFN2 structures, atomic force microscopy (AFM) was performed. AFM imaging and height analysis confirmed that the DFN2 nanoclusters are predominantly sub-2 nm in size, consistent with the expected dimensions of DNA-stabilized silver nanoclusters (Figure 1d-f).^2,33^ The apparent difference between DLS and AFM measurements likely arises from the inherent sensitivity of DLS to larger assemblies or aggregates in solution, which can influence the measured hydrodynamic diameter. Because DNA-templated silver nanoclusters are often prone to fluorescence decay over time, we systematically evaluated the stability of this formulation at multiple time points, including immediately following synthesis (∼1 h), 1 day, 7 days, and up to 48 days of storage, Figure 1i-j. The results indicate that the nanoclusters undergo an aging process, reaching optimal fluorescence intensity after approximately 24 h. This enhanced fluorescence remains stable for at least 48 days when stored under dark conditions at room temperature, demonstrating excellent shelf life and suitability for long-term biosensing applications.

**Figure 1:**
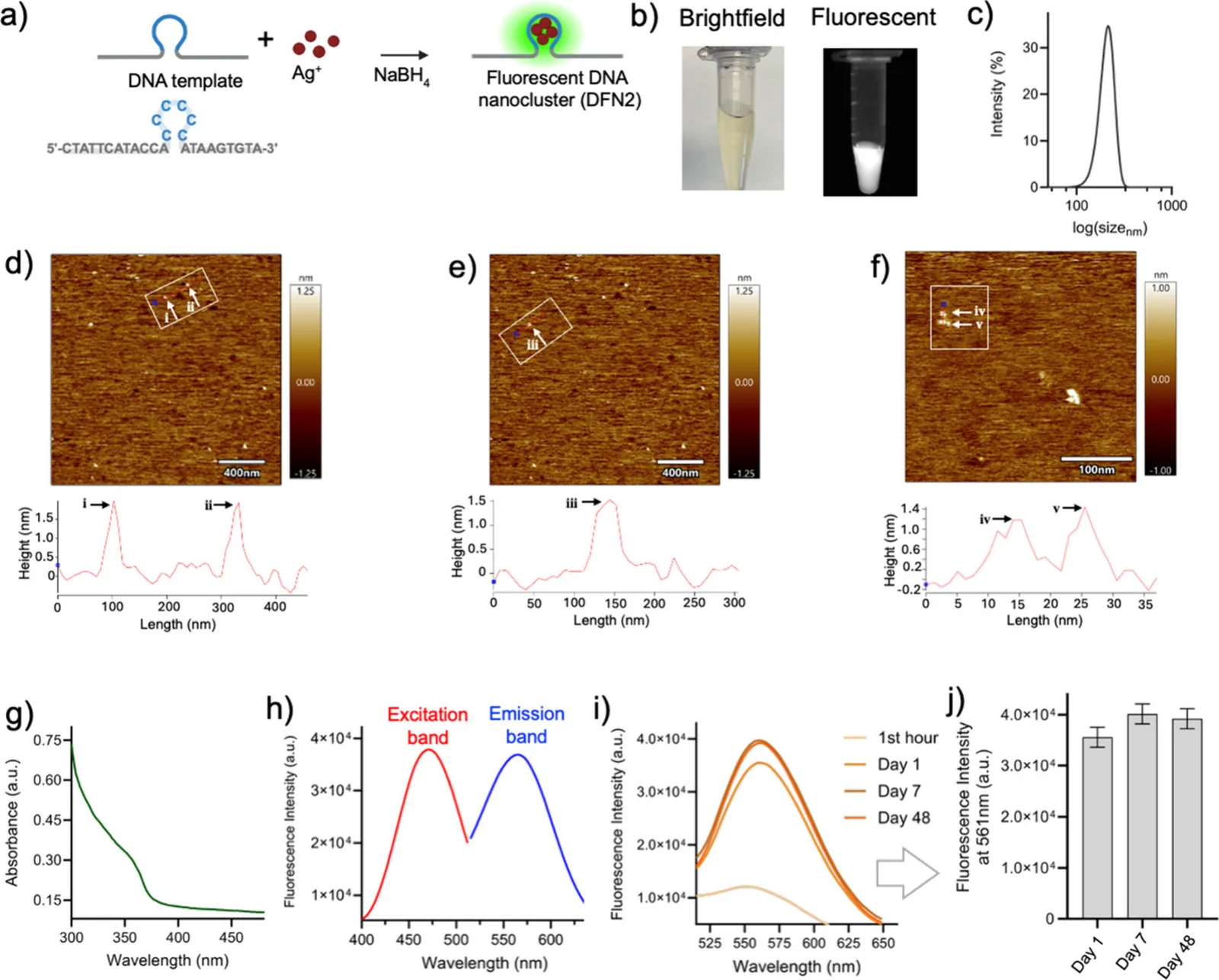
Synthesis and optical characterization of DFN2 nanoclusters. (a) DNA-templated fluorescent nanoclusters (DFN2) were synthesized using a 27-nt single-stranded DNA containing a central cytosine-rich region for Ag^+^ coordination. (b) Incubation with silver ions in the presence of a reducing agent produced a fluorescent DNA-Ag nanocluster complex exhibiting a characteristic light-brown colloidal appearance. (c) Dynamic light scattering (DLS) measurements of the prepared DFN2 mixture. (d–f) AFM images of individual DFN2 structures; height-profile analysis confirms the presence of sub-2 nm nanoclusters. (g) UV–vis spectroscopy revealed absorption between 300–400 nm. (h) Fluorescence characterization showed an excitation band (∼471 nm) and an emission peak at ∼561 nm. (i,j) Time-dependent fluorescence stability studies demonstrated an aging-dependent enhancement in fluorescence, reaching maximum intensity after ∼24 h and remaining stable for up to 48 days under dark storage conditions. Readings were collected in 96-well plates. Experiments were performed in triplicate, and data are presented as mean ± SD.

Following physicochemical characterization, we investigated the response of this nanocluster (DFN2) to nucleic acid hybridization. Initially, the 27-nt DNA template was prehybridized with complementary 27-nt DNA or RNA strands to form duplex structures with a central C-rich loop (Figure S1). These DNA:DNA and DNA:RNA complexes were then used as templates for silver nanocluster synthesis to evaluate whether prehybridization affects fluorescence generation. Both complexes produced negligible fluorescence at 561 nm, comparable to the buffer control lacking DNA template (C6), Figure 2b, indicating that the duplexed templates do not support development of fluorescence in nanoclusters.

**Figure 2:**
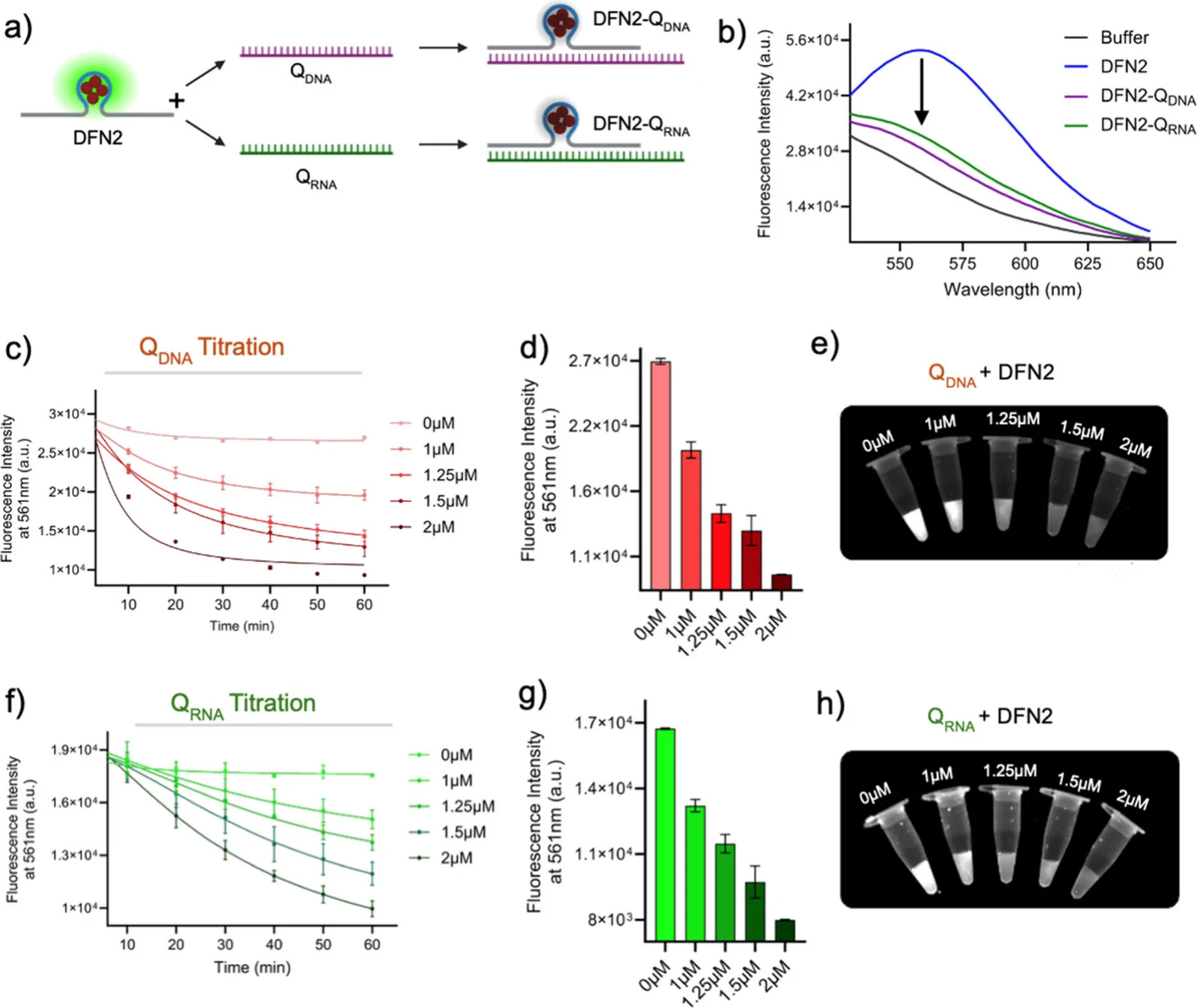
Hybridization-mediated fluorescence quenching of DFN2. Hybridization of DFN2 with complementary DNA or RNA strands was investigated. (b) Prehybridized DNA:DNA and DNA:RNA templates containing a central C-loop were used for nanocluster formation and exhibited negligible fluorescence at ∼560 nm, comparable to buffer controls. In contrast, DFN2 synthesized on single-stranded templates showed strong fluorescence. Addition of complementary (c) quencher DNA (Q_DNA_) or (f) quencher RNA (Q_RNA_) resulted in concentration-dependent fluorescence quenching over time, confirming that hybridization suppresses DFN2 emission through structural regulation of the DNA template. (d, g) End point fluorescence intensity at 561 nm after 60 min incubation. (e, h) Representative fluorescence imaging showing concentration-dependent quenching for Q_DNA_ and Q_RNA_, respectively. Readings were collected in 96-well plates. Experiments were performed in triplicate, and data are presented as mean ± SD.

To systematically investigate hybridization-induced fluorescence modulation in a context relevant to CRISPR-based biosensing applications, DFN2 nanoclusters were first synthesized using the single-stranded DNA template and subsequently titrated with complementary strands, termed quencher DNA (Q_DNA_) and quencher RNA (Q_RNA_), Figure 2a. As shown in Figure 2c, increasing concentrations of Q_DNA_ resulted in real-time fluorescence quenching, with faster kinetics observed at higher quencher DNA concentrations over a 60 min measurement period. End point quantitative measurements, along with qualitative fluorescence imaging using a benchtop digital imaging platform, demonstrated a progressive decrease in DFN2 fluorescence intensity with increasing Q_DNA_ concentration (Figure 2d-e and S3a).

A similar set of experiments was performed using Q_RNA_ strands. Consistent with the Q_DNA_ results, Q_RNA_ strands also induced a concentration-dependent decrease in fluorescence over time (Figure 2f). Although the DFN2-Q_RNA_ system exhibited slower quenching kinetics compared to DFN2-Q_DNA_, the overall trend supports a hybridization-mediated fluorescence regulation mechanism. End point measurements at 561 nm and qualitative fluorescence imaging further confirmed a concentration-dependent reduction in signal, Figure 2g-h and Figure S3b.

After confirming that hybridization suppresses the fluorescence of DFN2, we next investigated whether this fluorescence could be restored using invading DNA or RNA strands fully complementary to the quencher strands. In the quenched DFN2-Q_DNA_ and DFN2-Q_RNA_ complexes, hybridization is incomplete, leaving the cytosine-rich region partially unpaired, (Figure S1). In contrast, the invading strands (Inv_DNA_ and Inv_RNA_) are fully complementary to the quencher strands (Figure S2) and are therefore expected to displace them from the DFN2 complexes via strand displacement and competitive hybridization, (Figure 3a and Figure S2b-e).

**Figure 3:**
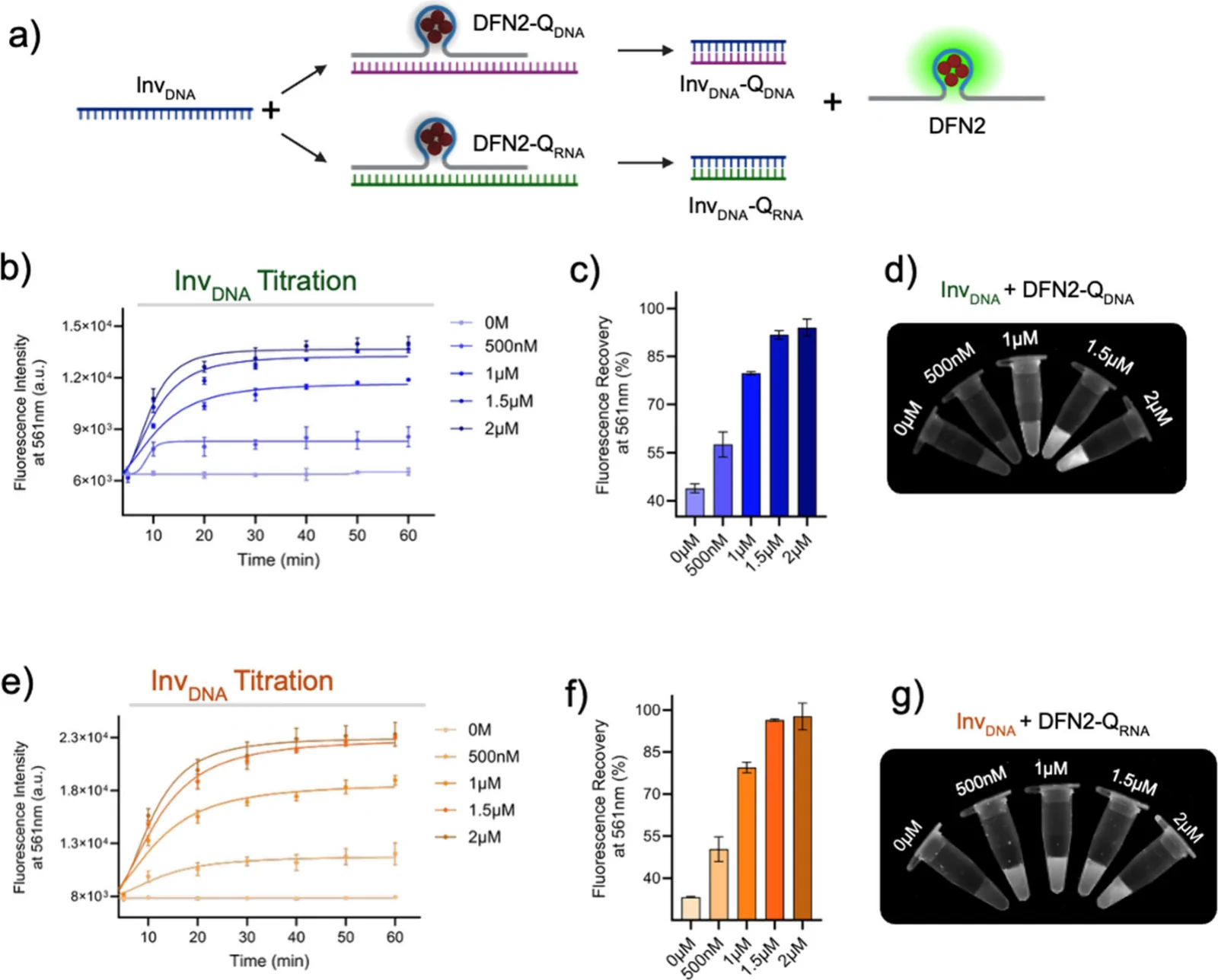
Reversible fluorescence switching via invasive DNA strand displacement. (a) Fluorescence recovery of quenched DFN2 systems was evaluated using invading DNA strand complementary to the quencher sequences. (b) Invading DNA (Inv_DNA_) restored fluorescence in DFN2-Q_DNA_ complexes in a concentration-dependent manner via strand displacement. (e) Similarly, Inv_DNA_ efficiently displaced quencher RNA from DFN2–Q_RNA_ complexes, recovering fluorescence. In both cases, (c, f) end point percent fluorescence recovery after 60 min incubation and (d, g) fluorescence imaging confirmed reversible ON-OFF-ON switching behavior, demonstrating programmable and reversible regulation of DFN2 fluorescence. Readings were collected in 96-well plates. Experiments were performed in triplicate, and data are presented as mean ± SD.

To test this, DFN2-Q_DNA_ complexes were titrated with Inv_DNA_. Remarkably, fluorescence recovery was observed over time, with greater restoration at higher Inv_DNA_ concentrations. End point measurements and fluorescence imaging confirmed that the quenched signal could be effectively recovered through this strand displacement mechanism (Figure 3c,d and Figure S4a). These results demonstrate that the initially quenched DFN2 complex can regain its fluorescence upon removal of the quencher strands.

We then performed analogous experiments using DFN2-Q_RNA_ complexes. Similarly, Inv_DNA_ was able to displace the quencher RNA strands in a concentration-dependent manner, leading to reproducible fluorescence recovery (Figure 3e). End point quantitative measurements and fluorescence imaging further supported this behavior (Figure 3f,g and Figure S4b). This reversible ON-OFF-ON fluorescence switching is highly advantageous for biosensing applications demonstrating that DFN2 operates as a dynamically and precisely programmable optical system governed by nucleic acid hybridization.

Following these studies, we further investigated whether an invading RNA strand (Inv_RNA_) could similarly induce strand displacement and disrupt the hybridization within DFN2-Q_DNA_ and DFN2-Q_RNA_ complexes, thereby restoring fluorescence, Figure 4a. Consistent with the Inv_DNA_ results, Inv_RNA_ successfully recovered fluorescence in both systems demonstrating the robustness, reproducibility, and predictability of this platform, Figure 4b-g and Figure S5. Such reversible and well-controlled behavior of these DNA-templated silver nanoclusters brings renewed attention to the field, as neither the DNA template nor the quencher strands require fluorescent or quenching labels. Instead, the system mimics the behavior of conventional fluorophore-quencher-labeled DNA/RNA constructs, while offering a significantly simpler and more cost-effective, label-free alternative.

**Figure 4:**
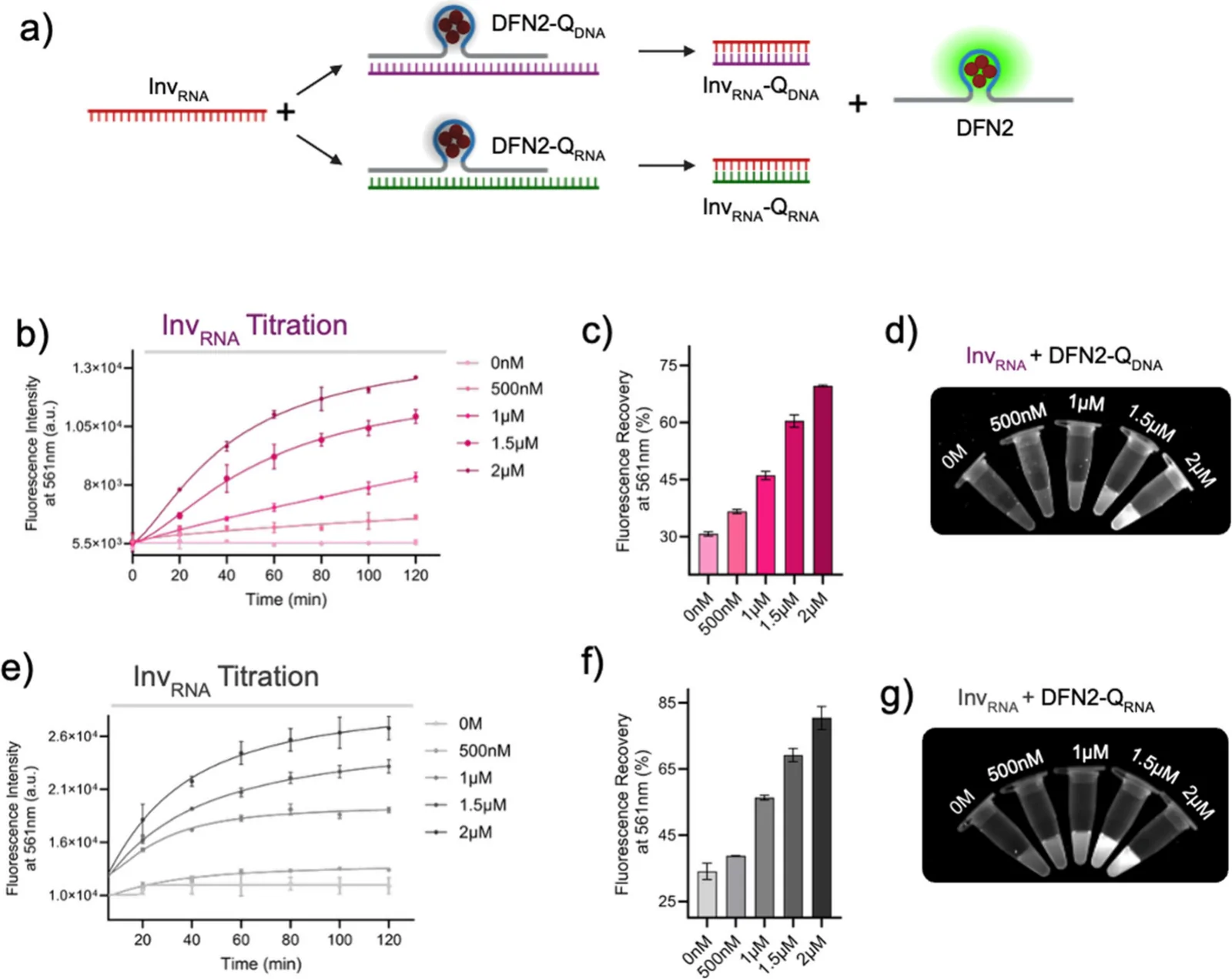
RNA-mediated strand displacement and hybridization regulation. (a) Fluorescence recovery of quenched DFN2 systems was evaluated using invading RNA strand complementary to the quencher sequences. (b) Invading RNA (Inv_RNA_) restored fluorescence in DFN2–Q_DNA_ complexes in a concentration-dependent manner via strand displacement. (e) Similarly, Inv_RNA_ efficiently displaced quencher RNA from DFN2-Q_RNA_ complexes, recovering fluorescence. In both cases, (c, f) end point percent fluorescence recovery after 120 min incubation and **(d**, **g)** fluorescence imaging confirmed reversible ON-OFF-ON switching behavior, demonstrating programmable and reversible regulation of DFN2 fluorescence. Readings were collected in 96-well plates. Experiments were performed in triplicate, and data are presented as mean ± SD.

After carefully characterizing the DFN2 system in its fluorescent “ON” state, as well as its hybridization-induced “OFF” state and strand displacement-mediated fluorescence recovery (“ON”), we next investigated whether this robust and reversible behavior could be leveraged for biosensing applications. Specifically, we integrated the system with an upstream CRISPR-cas12a-mediated mechanism to achieve a target-triggered fluorescence turn-on response. As a proof-of-concept, we examined activation of the Cas12a system using a conserved genomic region from the foodborne pathogen *Listeria monocytogenes*.

In this scheme, target recognition activates the Cas12a-crRNA complex, triggering its collateral cleavage activity. Upon activation, Cas12a degrades a designed regulator DNA strand (Reg_DNA_), which would otherwise bind and sequester the invading RNA strand (Inv_RNA_), Figure 5a. In the absence of Reg_DNA_, Inv_RNA_ is free to interact with the DFN2-Q_DNA_ complex, where it displaces the quencher DNA (Q_DNA_) through strand exchange, forming an Inv_RNA_-Q_DNA_ duplex. This process releases DFN2 and restores its fluorescence signal. Therefore, in the presence of the target, fluorescence is turned ON. Conversely, in the absence of the target, the Cas12a-crRNA complex remains inactive and Reg_DNA_ is not degraded, (Figure 5b). Under these conditions, Reg_DNA_ binds to Inv_RNA_, forming a stable complex that prevents Inv_RNA_ from interacting with the DFN2-QD_NA_ system. As a result, the quenched DFN2-Q_DNA_ complex remains intact, and fluorescence stays in the OFF state.

**Figure 5:**
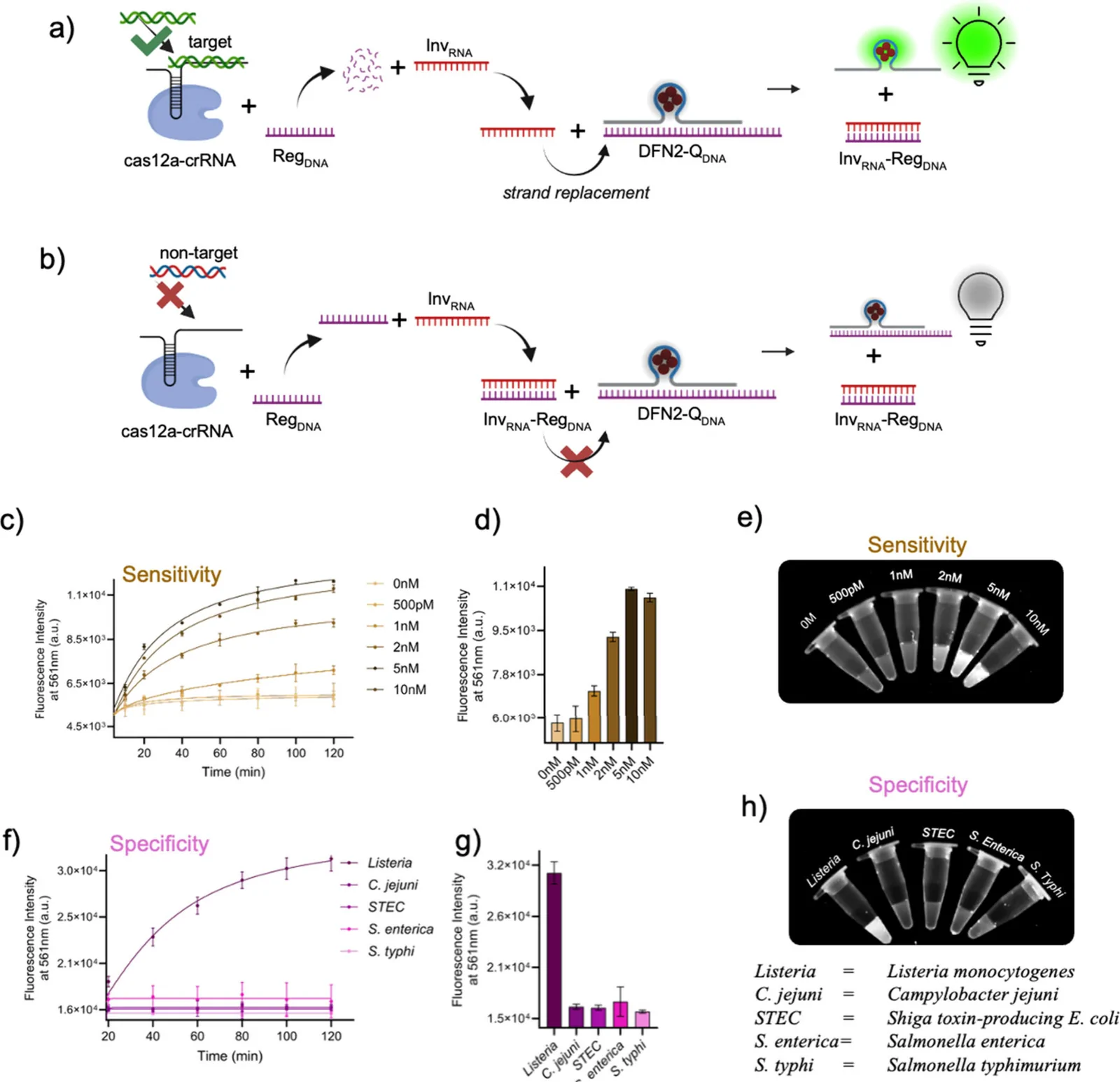
CRISPR–Cas12a-controlled biosensing using DFN2 nanoclusters. A CRISPR–Cas12a-mediated biosensing platform was constructed by integrating DFN2 with a strand displacement circuit. (a) Upon recognition of a conserved genomic target, the activated Cas12a–crRNA complex cleaves a regulatory DNA strand (Reg_DNA_), thereby preventing sequestration of the invading RNA strand (Inv_RNA_). The free Inv_RNA_ displaces the quencher strand (Q_DNA_) from DFN2–Q_DNA_, restoring fluorescence (ON state). (b) In the absence of the target, Reg_DNA_ remains intact, sequestering Inv_RNA_ and maintaining the OFF state. (c) The system exhibits concentration- and time-dependent fluorescence responses, enabling detection down to 1 nM without amplification. (d) End point fluorescence measurements and (e) fluorescence imaging after 2 h. (f) Specificity tests against target genomic regions from STEC, *Campylobacter jejuni*, *Salmonella typhi*, and *Salmonella enterica* show negligible cross-reactivity, confirming high target selectivity. (g) End point fluorescence measurements and (h) fluorescence imaging after 2 h. Readings were collected in 96-well plates. Experiments were performed in triplicate, and data are presented as mean ± SD.

To evaluate this mechanism, we first designed a crRNA targeting a conserved genomic fragment of *Listeria monocytogenes* and tested the Cas12a-crRNA-initiated DFN2 assay across varying target concentrations (Figure 5c). As the target concentration increased, fluorescence intensity progressively increased over time, exhibiting both concentration-dependent and time-dependent responses over a 2-h incubation period. End point analysis demonstrated that target concentrations as low as 1 nM (6.02 × 10^8^ copies per μL) could be detected without any prior amplification (Figure 5d and Figure S6a). Fluorescence imaging further confirmed a clear, concentration-dependent increase in signal intensity (Figure 5e). Although the system without an amplification step is less sensitive than prior Cas12a-based detection assays,^19–22^ it is inexpensive, rapidly prepared, and does not require chemical labeling, making it attractive for low-cost and accessible point-of-care applications. For food screening, ultrahigh sensitivity is not always a concern, as excessively sensitive assays may detect contamination levels below practical or regulatory thresholds.

To assess the specificity of the assay, we tested Cas12a-crRNA, programmed for *Listeria*, and DFN2 assay against conserved genomic regions from Shiga toxin-producing *Escherichia coli* (STEC), *Campylobacter jejuni*, *Salmonella typhi*, and *Salmonella enterica*. As shown in Figure 5f, none of the nontarget sequences produced a measurable increase in fluorescence, whereas the target genomic fragment generated a fluorescence recovery, Figure 5f-h. These results confirm the high specificity of the system, attributed to programmable Cas12a activation guided by the crRNA.

In summary, we systematically investigated the hybridization and strand displacement behavior of a programmable DNA-templated silver nanocluster system and established a versatile, label-free fluorescence platform with reversible optical switching. The nanoclusters exhibit strong emission at 561 nm and undergo efficient and predictable fluorescence quenching upon duplex formation with complementary DNA or RNA strands. Importantly, the fluorescence signal can be fully restored through invading strand-mediated displacement, enabling robust and reversible ON-OF-ON optical control. By integrating this nanocluster platform with a CRISPR-Cas12a, we developed a modular, enzyme-coupled sensing architecture in which target recognition initiates a cascade of strand displacement reactions that ultimately restore fluorescence output though and invasive RNA. Unlike our previous work using a different DNA-templated nanocluster system that generated a turn-off signal and required a two-step Cas12a process for turn-on signal, the present platform enables a controllable turn-on signal in a single step.^33^ This simplified and robust design highlights the potential of these responsive, label-free DNA-templated nanoclusters as powerful tools for biosensing applications. This strategy enables detection at the nanomolar level without nucleic acid amplification and maintains high specificity against nontarget sequences. Overall, this work establishes DNA-templated silver nanoclusters as programmable, enzyme-responsive optical reporters and provides a generalizable framework for label-free biosensing platforms.

## Supplementary Material



## Data Availability

The data supporting the findings of this study are available within the article and its Supporting Information. Additional data are available from the corresponding author upon reasonable request.

## Notes

Detailed experimental procedures and calculated DNA-RNA hybridization analyses using NUPACK, additional experimental details, materials lists, and oligonucleotide sequences (PDF)
