# Supplementary material for: Invasive RNA Programs Optical Switching in DNA Nanoclusters for CRISPR Sensing
Source: Nano Lett. 2026 Aug 31;26(36):11988–95. doi: 10.1021/acs.nanolett.6c01946 (PMC13587672; doi:10.1021/acs.nanolett.6c01946)
Supplement: Supplementary file 1 [file nl6c01946_si_001.pdf]

Supporting Information

Invasive RNA Programs Optical Switching in DNA Nanoclusters for CRISPR Sensing

Reggie Gold[†,‡], Yusha Imtiaz[†], Vinod Morya[‡], Ken Halvorsen[‡], Emmett Hanson[†], and Mehmet V. Yigit[*,†,‡]

† Department of Chemistry,
University at Albany, State University of New York,
1400 Washington Avenue, Albany, New York 12222, United States.

‡ The RNA Institute,
University at Albany, State University of New York,
1400 Washington Avenue, Albany, New York 12222, United States.

*Correspondence:

Tel: (1) 518-442-3002

myigit@albany.edu

Materials and Methods.

Materials. All DNA and RNA oligonucleotides were purchased from Integrated DNA Technologies, INC. (Coralville, IA, USA) **(Table S1)**. Chemicals including Tris Base ($NH_2C(CH_2OH)_3$; CAS Number: 77-86-1), EDTA ($(HO_2CCH_2)_2NCH_2CH_2N(CH_2CO_2H)_2$; CAS Number: 60-00-4), Magnesium Acetate Tetrahydrate ($(CH_3COO)_2Mg \cdot 4H_2O$; CAS Number: 16674-78-5), Silver Nitrate ($AgNO_3$; CAS Number: 7761-88-8), and Sodium Borohydride ($NaBH_4$; CAS Number: 16940-66-2), were purchased from Sigma-Aldrich (St. Louis, MO, USA). Acetic Acid (CH_3CO_2H; CAS Number: 64-19-7) was purchased from Honeywell Fluka (Seezle, Germany). EnGen Lba Cas12a (Cpf1) (Catalog Number: M0653T) was sourced from New England BioLabs Inc. (Ipswich, MA, USA). Molecular grade water (Catalog Number: 786-292) was purchased from G-Biosciences (St. Louis, MO, USA). Costar 96-well half-area microwell plates (Catalog Number: 29444-316) were acquired from VWR International (Seacaucus, NJ, USA). The *Listeria monocytogenes* EGD-e genome (Catalog Number: BAA-679D-5, Batch Number: 70071410) was purchased from the American Type Culture Collection (ATCC; Manassas, VA, USA).

Equipment and Software. Ultraviolet–visible (UV–Vis) spectroscopy measurements were performed using an Agilent Technologies Cary 60 UV-Vis spectrophotometer (Part No. G6960AA). Fluorescence measurements were acquired with an Agilent Technologies BioTek Synergy H1 multimode reader. All incubations were carried out using a Bio-Rad T100™ thermal cycler. Fluorescence imaging was conducted with a Bio-Rad ChemiDoc™ MP imaging system. Dynamic light scattering (DLS) measurements were obtained using a Malvern Zetasizer Nano series instrument. Data analysis and figure preparation were performed using GraphPad Prism. Schemes and mechanistic illustrations were generated using BioRender (biorender.com).

Methods.

Oligonucleotide Preparation. All DNA and RNA oligonucleotides were dissolved in molecular-grade water to prepare 1 mM stock solutions. Serial dilutions for individual experiments were prepared in the corresponding experimental buffers. Oligonucleotide concentrations were determined by UV–Vis spectroscopy using molar extinction coefficients provided in the manufacturer's certification sheets.

Buffer Preparation. The following buffers were prepared using molecular grade water. After preparation, all buffers were sterilized with an autoclave liquid cycle to remove contaminants.

Synthesis Buffer: 40mM Tris Base, 30mM Acetic Acid, 12.5mM Magnesium Acetate Tetrahydrate, and 2mM EDTA, pH 7.5

DFN2 Reaction Buffer: 40mM Tris Base, 22.5mM Acetic Acid, 12.5 mM Magnesium Acetate Tetrahydrate, 2mM EDTA, pH 8.0

DNA-Templated Fluorescent Silver Nanoclusters (DFN2) Synthesis. To obtain DNA-templated fluorescent silver nanoclusters (DFN2), 100µL of 100µM DFN2 synthesis DNA (C6, **Table S1**), 6µL of 10µM $AgNO_3$, and 840µL of *Synthesis Buffer* were lightly mixed in a 1.5mL microcentrifuge tube. After incubation for 3 hours at 4°C in dark conditions, 6µL of 10mM $NaBH_4$ was added. The solution was immediately vortexed for 5 minutes until reaching a pale-yellow hue and stored at 4°C in the dark for 24 hours before use.

Preparation of Quenched Fluorescent Silver Nanoclusters (DFN2) Following RNA and DNA Hybridization. To obtain DNA and RNA-quenched silver nanoclusters (DFN2-Q_{DNA} and DFN2-Q_{RNA}, respectively), 10µL of 100µM C6 was combined with either 10µL of 100µM DNA-Quencher (Q_{DNA}) or RNA-Quencher (Q_{RNA}) and 80µL of Synthesis Buffer. This solution was heated to 95°C for 5 minutes, then allowed to cool slowly on benchtop for 3 hours, and cool for a final 3 hours at 4°C. 6µL of 1mM $AgNO_3$ and 0.6µL of 10mM $NaBH_4$ was added before vortexing for 5 minutes. DFN2-Q_{DNA} and DFN2-Q_{RNA} were incubated at 4°C for 24 hours before use.

Note: $AgNO_3$ and $NaBH_4$ were stored at 4°C in airtight and light sensitive containers to prevent degradation. $NaBH_4$ solution was made fresh during each synthesis and added within 30 seconds. All nanoclusters were stored at 4°C in dark conditions when not used.

DFN2 (Fluorescent DNA Nanocluster) Characterization. Absorbance and fluorescent spectra were recorded with 10µL DFN2, DFN2-Q_{DNA}, or DFN2-Q_{RNA}, and 40µL molecular grade water, in a 96-well half area-plate. A hydrodynamic radius of DFN2 after 100x dilution in molecular grade water. DFN2, DFN2-Q_{DNA}, or DFN2-Q_{RNA} were fluorescently imaged 24 hours following synthesis with blue illumination and a 532/28 emission filter.

Hybridization-Induced Quenching of DFN2 with Complementary Quencher DNA and RNA strands. 9.52µL of stock DFN2 (10.5µM) were added to 35.48µL reaction buffer, to reach a final concentration of 2µM in solution. After five minutes, 5µL of Q_{DNA} or Q_{RNA} were added to the solution to obtain various final concentrations of quencher from 1µM to 2µM. The fluorescence of the solution was monitored over 1 hour at an experimental temperature of 25°C with excitation and emission parameters of 471nm and 561nm, respectively.

Strand Replacement-Induced Unquenching (Fluorescence Recovery) Using Invading DNA Strand ($\mathbf{Inv_{DNA}}$). 9.52µL of stock DFN2-Q_{DNA} or DFN2-Q_{RNA} (10.5µM) were added to 35.48µL reaction buffer, to reach a final concentration of 2µM in solution. After five minutes, 5µL of a DNA invading strand (Inv_{DNA}) was added to the solution to obtain various final concentrations from 500nM to 2µM. The experimental temperature was maintained at 25°C, and fluorescence of the solution was monitored over 1 hour at an excitation and emission of 471nm and 561nm, respectively.

Strand Replacement-Induced Unquenching (Fluorescence Recovery) Using Invading RNA Strand ($\mathbf{Inv_{RNA}}$). 9.52µL of DFN2-Q_{DNA} (10.5µM) were added to 35.48µL reaction buffer, to reach a final concentration of 2uM in solution. After five minutes, 5µL of a RNA invading strand (Inv_{RNA}) was added to

the solution to obtain various final concentrations from 500nM to 2μM. The fluorescence of the solution was monitored over 1 hour at 25°C with an excitation and emission of 471nm and 561nm, respectively.

Designing of crRNA and Truncated Targets. A conserved target region within the *hly* gene of the *Listeria monocytogenes* EGD-e genome was identified using SnapGene (snapgene.com) and the NCBI nucleotide BLAST tool. A double-stranded DNA (dsDNA) sequence containing a TTTN protospacer adjacent motif (PAM) and a high GC content was selected and designated as a truncated dsDNA sequence (dsListeria). A corresponding guide RNA (crRNAListeria) was designed using the Integrated DNA Technologies (IDT) design platform to ensure compatibility with CRISPR–Cas12a. Additional truncated DNA sequences were designed following a similar approach, in accordance with previously reported procedures.[1]

CRISPR-Cas12a Induced TURN-ON Detection Using DFN2 Nanoclusters. A CRISPR-Cas12a complex was first prepared by combining the Cas12a enzyme with a Listeria specific CRISPR-guide RNA ($crRNA_{Listeria}$) in 1:1.2 ratio in DFN2 reaction buffer to reach final concentrations of 1μM and 1.2μM in 60μL, respectively. This complex was then incubated at 37°C for 30 minutes to promote complex formation. Following this, 5μL of this complex (final concentration of 100nM) was added to 5μL of truncated-Listeria dsDNA ($dsDNA_{Listeria}$) at final concentrations ranging from 500pM to 10nM in 50μL. After a second incubation period of 30 minutes at 37°C, 5μL of a regulator DNA (Reg_{DNA}) (final concentration 2μM), which is prone to cas12a cleavage, was added and left to incubate for a final period of 1 hour at 37°C. When complete, 5μL of Inv_{RNA} (final concentration 2μM) and 20.48μL of DFN2 reaction buffer were added. This reaction mixture was then heated to 65°C for 15 minutes to inhibit any CRISPR-Cas12a enzymatic activity before cooling to room temperature. The cooled mixture was added to 9.52μL of DFN2-Q_{DNA}, where the fluorescence was monitored over 2 hours at 25°C with an excitation and emission of 471nm and 561nm, respectively.

Specificity of The TURN-ON Assay. A CRISPR-Cas12a complex was prepared containing Cas12a enzyme and $crRNA_{Listeria}$ in DFN2 reaction buffer as described above. Following an incubation period of 30 minutes at 37°C, 5μL of truncated control dsDNAs targets ($dsDNA_{C.\ jejuni}$, $dsDNA_{STEC}$, $dsDNA_{S.\ enterica}$

and $dsDNA_{S.\ typhi}$) were added to 5µL of the CRISPR-Cas12a complex and allowed to incubate for 30 additional minutes at 37°C. 5µL of Reg_{DNA} (final concentration 2µM) was added and left to incubate for a final period of 1 hour at 37°C. When complete, 5µL of Inv_{RNA} (final concentration 2µM) and 20.48µL of DFN2 reaction buffer were added. This reaction mixture was then heated to 65°C for 15 minutes to inhibit any CRISPR-Cas12a enzymatic activity before cooling to room temperature. The cooled mixture was added to 9.52µL of DFN2-Q_{DNA}, where the fluorescence was monitored over 2 hours at 25°C with an excitation and emission of 471nm and 561nm, respectively.

Atomic Force Microscopy Studies. a Cypher-S Atomic Force Microscope (Asylum Research, Oxford Instruments) with a liquid droplet probe holder and a BL-AC40TS probe was operated in tapping (AC) mode. Freshly cleaved mica substrates were prepared, and 5 µL of DFN-2 sample was deposited onto the mica surface and allowed to incubate for 1 min. Subsequently, 100 µL of 1x TAE-Mg buffer was added to the mica for imaging, and an additional drop of the same buffer was placed on the cantilever tip prior to mounting the probe on the sample stage for imaging.

References.

(1) Hanson, E.; Gold, R.; Lee, D. H.; Yigit, M. V. Cas12a-Programmed Modular CRISPR Cascade Reaction on Paper Supports for Dual-Mode Detection of Pathogenic Genomes. *ACS Sens.* 2026, *11* (3), 2774–2783.

Table S1. DNA and RNA Oligonucleotide Sequences.

C6 (Synthesis DNA)	5’ CTATTCATACCACCCCCCATAAGTGTA 3’
Q_{DNA} (DNA Quenching Strand)	5’ ATGTCAGACACTTATTGGTATGAATAG 3’
Q_{RNA} (RNA Quenching Strand)	5’ AUGUCAGACACUUAUUGGUAUGAAUAG 3’
Inv_{DNA} (Invading DNA Strand)	5’ CTATTCATACCAATAAGTGTCTGACAT 3’
Inv_{RNA} (Invading RNA Strand)	5’ CUAUUCAUACCAAUAAGUGUCUGACAU 3’
Reg_{DNA} (Regulating DNA Quenching Strand)	5’ ATGTCAGACACTTATTGGTATGAATAG 3’
dsDNA $_{Listeria}$ (Truncated *Listeria monocytogenes* dsDNA target)	5' AAAGTAAAAGCTGCTTTTGATGCTGCCGTAAGCGGAAAATCTGTCTCAGG 3'<br>3' TTTCATTTTCGACGAAAACTACGACGGCATTCGCCTTTTAGACAGAGTCC 5'
$dsDNA_{C.\ jejuni}$ (Truncated *Campylobacter jejuni* dsDNA target)	5’ AGTTATTGGAAGGGGTGGTCATGGAAGTGCTCCAGAAAAGGCAAAAGATC 3’<br>3’ TCAATAACCTTCCCCACCAGTACCTTCACGAGGTCTTTTCCGTTTTCTAG 5’
$dsDNA_{STEC}$ (Truncated *Shiga Toxin-producing Escherichia coli* dsDNA target)	5’ TTTTTACCCTGTCCACACGATGCCAATGTACTCGGAAAAATATCAAAAGC 3’<br>3’ AAAAATGGGACAGGTGTGCTACGGTTACATGAGCCTTTTTATAGTTTTCG 5’
$dsDNA_{S.\ enterica}$ (Truncated *Salmonella enterica* ssDNA target)	5’ GGTATCTGCTGAAGTTGAGGATGTTATTCGCAAAGGGATCCGTCAGACCT 3’
$dsDNA_{S.\ typhi}$ (Truncated *Salmonella typhimurium* ssDNA target)	5’ TCGTCGTTAGGACTGATTGGCGATCTCGATAAAGTCTCTACAGAGACCGT 3’
$crRNA_{Listeria}$	5’ UAAUUUCUACUAAGUGUAGAUCGCUUACGGCAGCAUCAAAAGC 3’

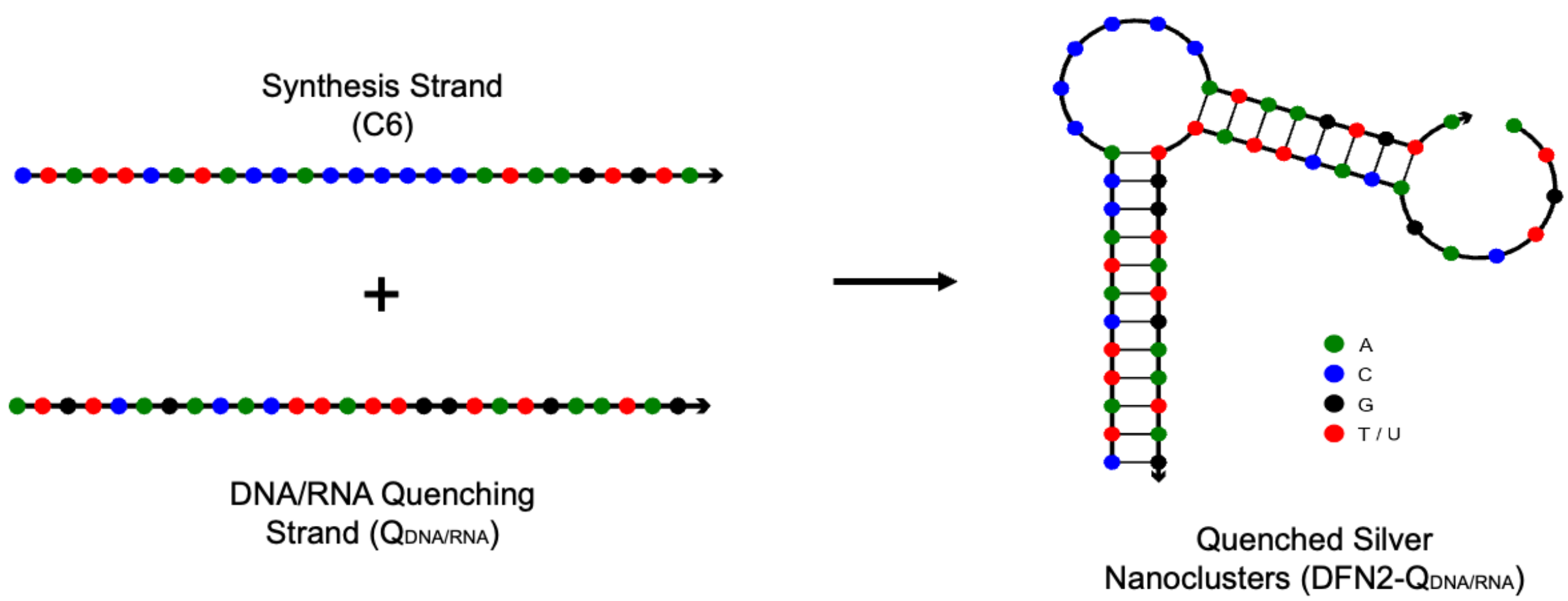

Figure S1. Hybridization-Mediated Fluorescence Quenching of DFN2. A single-stranded DNA "synthesis" strand (C6) is allowed to hybridize with a nearly complimentary (19 of 27bp) single-stranded DNA or RNA "quencher" strand (Q_{DNA} or Q_{RNA}, respectively). Upon addition of silver nitrate and reduction by sodium borohydride, quenched silver nanoclusters are achieved (DFN2-$Q_{DNA/RNA}$). The quenched nanoclusters are not complementary at the central 6-cytosine base region.

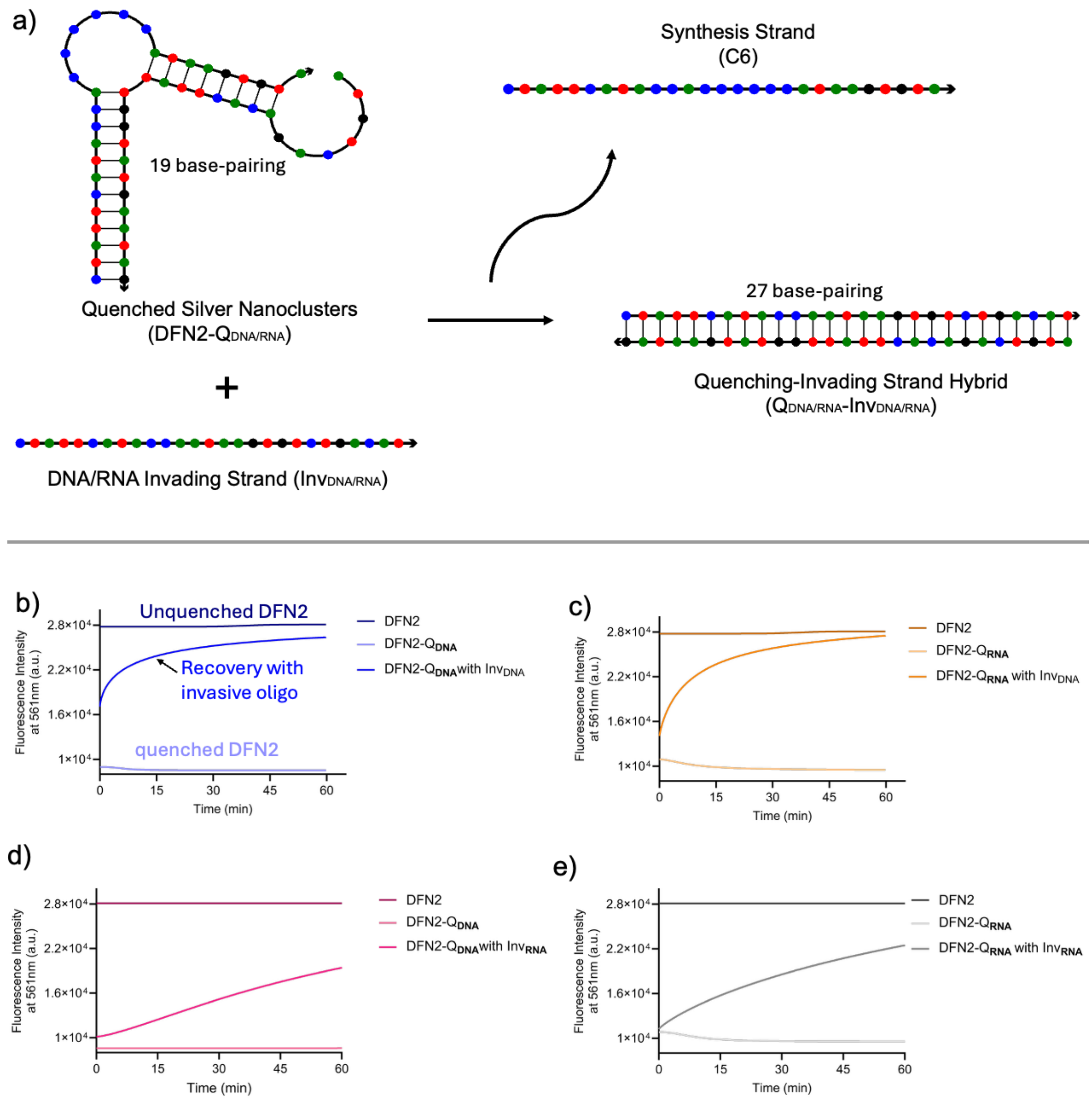

Figure S2. Fluorescent Recovery via an Invading DNA or RNA Oligonucleotide. Fluorescently quenched silver nanoclusters (DFN2-$Q_{DNA/RNA}$) are exposed to an invading DNA or RNA (Inv_{DNA} or Inv_{RNA}, respectively). **(a)** Strand replacement occurs creating a quenching-invading strand hybrid ($Q_{DNA/RNA}$-$Inv_{DNA/RNA}$) due to 27 base-pairing compared to 19 base-pairing of the quenched formulation. The strand displacement frees the synthesis strand and its central 6-cytosine base region, allowing a fluorescence recovery. Fluorescence intensities of unquenched DFN2, quenched DFN2, and fluorescence recovery following incubation with 2 µM invading oligonucleotide are shown for: **(b)** DFN2-Q_{DNA} + Inv_{DNA}, **(c)** DFN2-Q_{RNA} + Inv_{DNA}, **(d)** DFN2-Q_{DNA} + Inv_{RNA}, and **(e)** DFN2-Q_{RNA} + Inv_{RNA}.

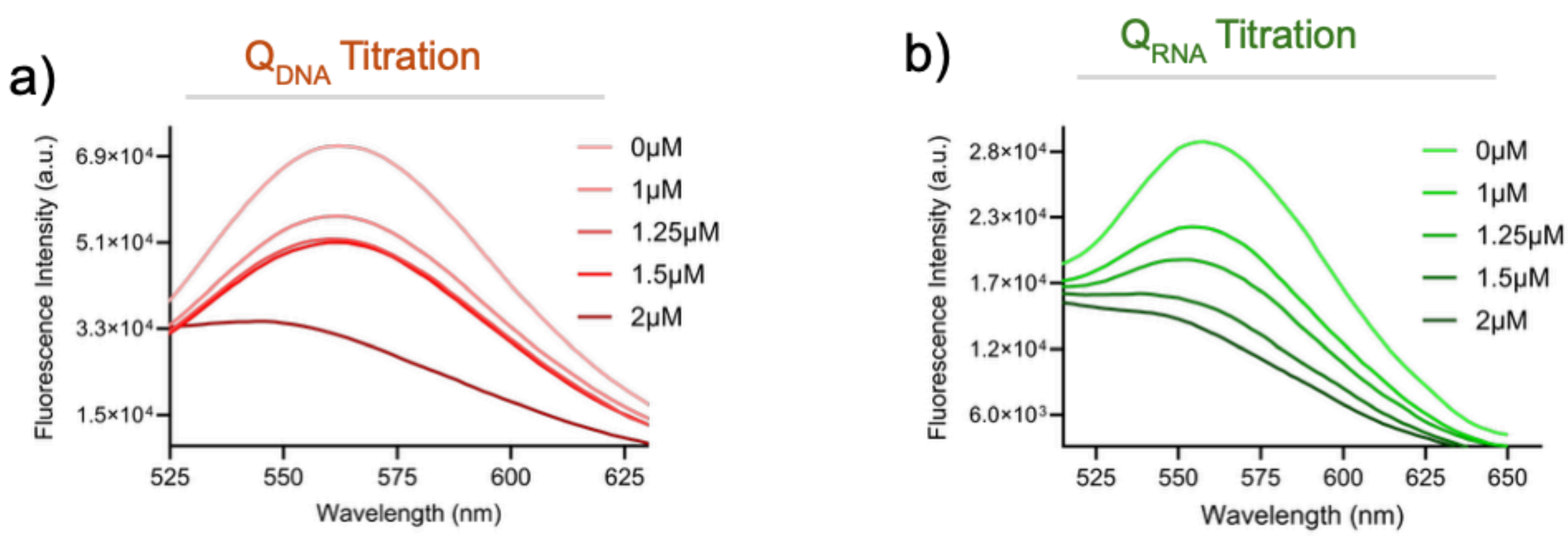

Figure S3. Endpoint Emission Spectra for Hybridization-Mediated Fluorescence Quenching of DFN2. Endpoint fluorescent spectra corresponding to **(a)** Figures 2c,d and **(b)** Figures 2f,g

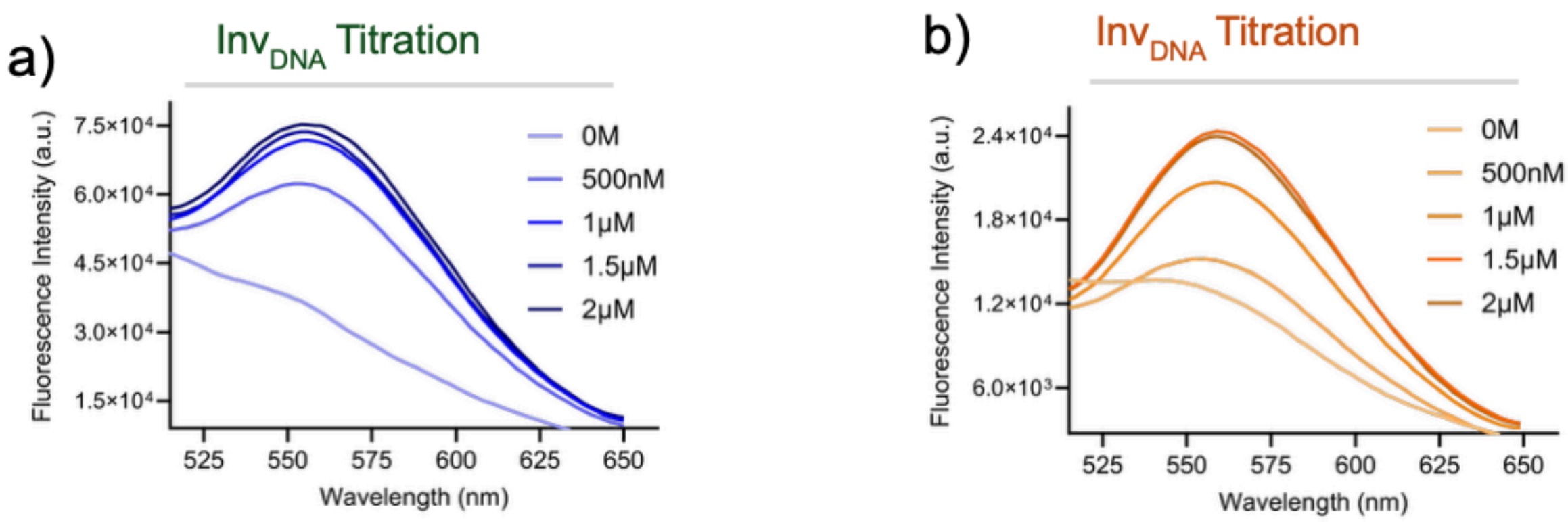

Figure S4. Endpoint Emission Spectra for Reversible Fluorescence Switching via Invasive DNA Strand Displacement. Endpoint fluorescent spectra corresponding to **(a)** Figures 3b,c and **(b)** Figures 3e,f.

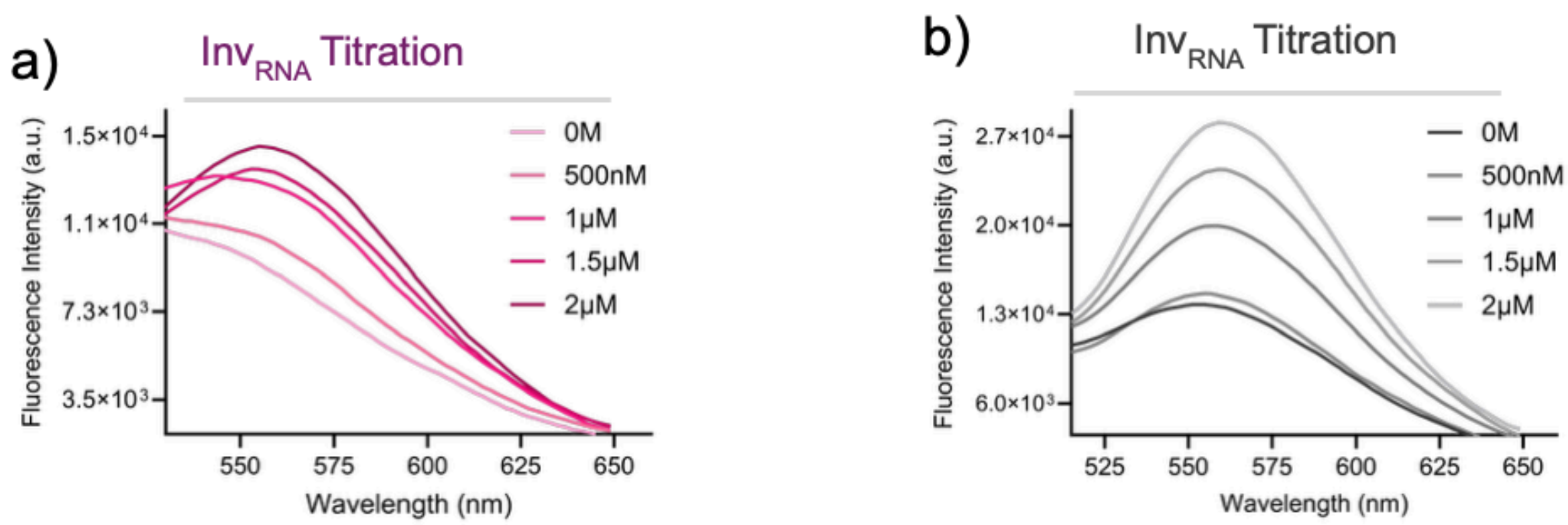

Figure S5. Endpoint Emission Spectra for RNA-Mediated Strand Displacement and Hybridization Regulation. Endpoint fluorescent spectra corresponding to **(a)** Figures 4b,c and **(b)** Figures 4e,f.

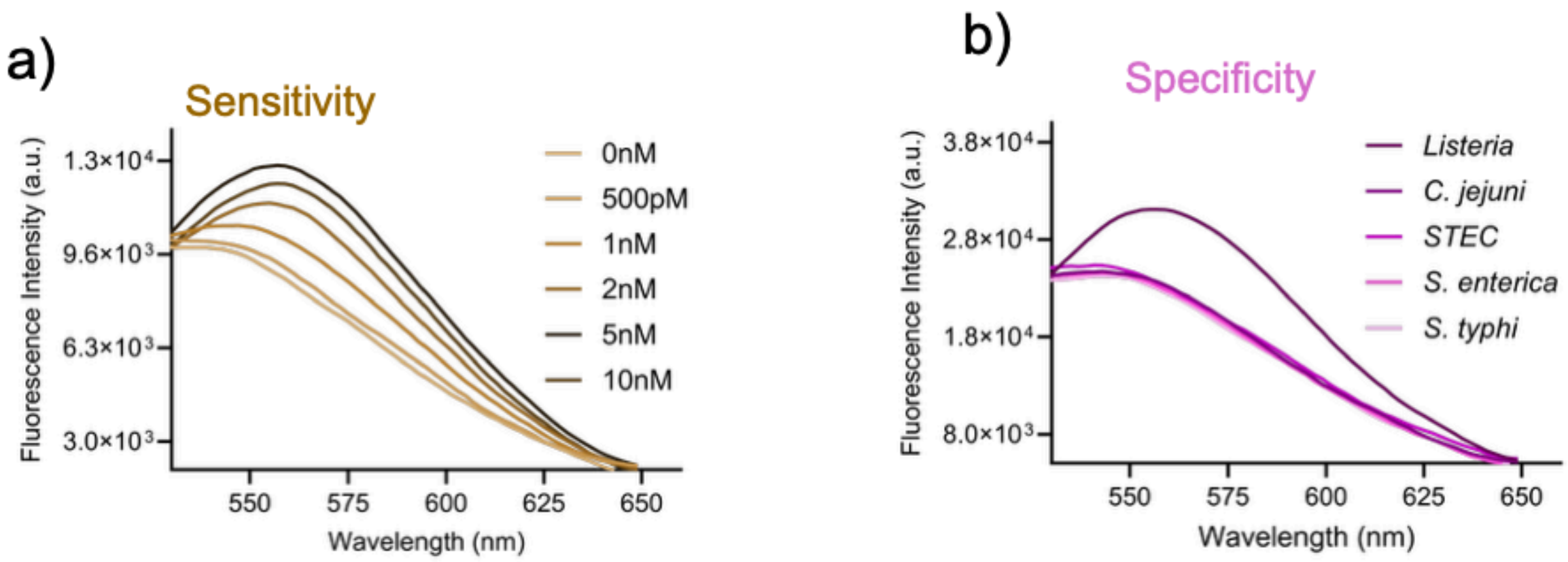

Figure S6. Endpoint Emission Spectra for CRISPR-Cas12a-Controlled Biosensing Using DFN2 Nanoclusters. Endpoint fluorescent spectra corresponding to **(a)** Figures 5c,d and **(b)** Figures 5f,g.
